# Retraction Note to: RIPK3 interactions with MLKL and CaMKII mediate oligodendrocytes death in the developing brain

**DOI:** 10.1038/s41419-022-05252-3

**Published:** 2022-10-07

**Authors:** Yi Qu, Jun Tang, Huiqing Wang, Shiping Li, Fengyan Zhao, Li Zhang, Q. Richard Lu, Dezhi Mu

**Affiliations:** 1 Department of Pediatrics, West China Second University Hospital, Sichuan University, Chengdu, 610041 China; 2 Key Laboratory of Birth Defects and Related Diseases of Women and Children (Sichuan University), Ministry of Education, Chengdu, 610041 China; 3 Department of Pediatrics, Division of Experimental Hematology and Cancer Biology, Cincinnati Children’s Hospital Medical Center, Cincinnati, 45229 OH USA; 4 Department of Pediatrics, University of California, San Francisco, CA 94143 USA

Retraction to: *Cell Death and Disease* 10.1038/cddis.2017.54, published online 23 February 2017

The Editors have retracted this article. Concerns have been raised regarding a number of figures, specifically:

- Figure 7a: there appear to be a number of repeating features in the bottom four panels.
- Figure 7b: there appear to be a number of repeating features in the bottom four panels.
- Figure 8b: there appear to be a number of repeating features in the panels for S, HI and RIPK3/Si for MPB(P21).

Additionally, the article shows significant overlap with a previously published article [1]. The Editors therefore no longer has confidence in the reliability of this article.

The authors have not responded to any correspondence from the editor/publisher about this retraction notice.
